# Redox regulation by TXNRD3 during epididymal maturation underlies capacitation-associated mitochondrial activity and sperm motility in mice

**DOI:** 10.1016/j.jbc.2022.102077

**Published:** 2022-05-25

**Authors:** Huafeng Wang, Qianhui Dou, Kyung Jo Jeong, Jungmin Choi, Vadim N. Gladyshev, Jean-Ju Chung

**Affiliations:** 1 Department of Cellular and Molecular Physiology, Yale School of Medicine, New Haven, Connecticut, USA; 2 Division of Genetics, Department of Medicine, Brigham and Women’s Hospital, Harvard Medical School, Boston, Massachusetts, USA; 3 Department of Biomedical Sciences, Korea University College of Medicine, Seoul, South Korea; 4 Department of Genetics, Yale School of Medicine, Yale University, New Haven, Connecticut, USA; 5 Department of Obstetrics, Gynecology, and Reproductive Sciences, Yale School of Medicine, New Haven, Connecticut, USA

**Keywords:** thioredoxin glutathione reductase, TXNRD3, redox homeostasis, male fertility, mitochondrial function, ultrastructure, AO, acridine orange, GPX4, glutathione peroxidase 4, HS, HEPES saline, HTF, human tubal fluid, OXPHOS, oxidative phosphorylation, TXNRD1, thioredoxin reductase 1, TXNRD2, thioredoxin reductase 2, TXNRD3, thioredoxin-glutathione reductase

## Abstract

During epididymal transit, redox remodeling protects mammalian spermatozoa, preparing them for survival in the subsequent journey to fertilization. However, molecular mechanisms of redox regulation in sperm development and maturation remain largely elusive. In this study, we report that thioredoxin-glutathione reductase (TXNRD3), a thioredoxin reductase family member particularly abundant in elongating spermatids at the site of mitochondrial sheath formation, regulates redox homeostasis to support male fertility. Using *Txnrd3*^*−/−*^ mice, our biochemical, ultrastructural, and live cell imaging analyses revealed impairments in sperm morphology and motility under conditions of TXNRD3 deficiency. We find that mitochondria develop more defined cristae during capacitation in *wildtype* sperm. Furthermore, we show that absence of TXNRD3 alters thiol redox status in both the head and tail during sperm maturation and capacitation, resulting in defective mitochondrial ultrastructure and activity under capacitating conditions. These findings provide insights into molecular mechanisms of redox homeostasis and bioenergetics during sperm maturation, capacitation, and fertilization.

Testicular sperm are functionally immature in that they lack the ability to naturally fertilize an egg. Epididymis transit is an indispensable step for mammalian sperm cells to fully develop the fertilizing ability. During epididymal descent, spermatozoa acquire the potential to develop progressive motility and capacitation (1, 2). One major threat to sperm for the subsequent journey to fertilization is oxidative damage since sperm plasma membrane is rich in polyunsaturated fatty acids (3). Redox mechanisms prepare epididymal spermatozoa for their protection and survival during the fertilization journey (4). For example, oxidation of the intracellular milieu is involved in mammalian sperm maturation and capacitation (5, 6). At the same time, oxidative damage correlates with lower motility of human sperm (7) and causes DNA fragmentation and protein crosslinking (8). However, it remains unclear how redox homeostasis is maintained during mammalian sperm capacitation.

Thioredoxin and glutathione systems are two major redox systems that utilize the thiol redox biology to maintain cellular redox homeostasis and protect against oxidative stress. Thioredoxin reductases, a family of selenium-containing pyridine nucleotide-disulfide oxidoreductases, are key redox regulators of mammalian thioredoxin system. They are comprised of three enzymes: thioredoxin reductase 1 (TXNRD1), thioredoxin reductase 2 (TXNRD2) (9, 10), and thioredoxin-glutathione reductase (TXNRD3, also known as TGR) (11, 12). TXNRD1 and TXNRD2 are essential proteins that support redox homeostasis in the cytosol and mitochondria, respectively (13, 14, 15, 16). TXNRD3 is predominantly expressed in testis (12) and particularly abundant in elongating spermatids at the site of mitochondrial sheath formation (17). Structurally, TXNRD3 contains an additional N-terminal glutaredoxin domain compared with the other two TXNRD isozymes (12). Thus, it has affinity to both thioredoxin and glutathione systems *in vitro* (12) and can reduce both. Disulfide bond isomerase activity of TXNRD3 was also previously reported *in vitro* (17), yet the physiological significance and underlying mechanisms of TXNRD3 in male germ cell development and sperm function have been largely unknown.

Here, we report that targeted disruption of *Txnrd3* in mice induces capacitation-associated impairment in sperm morphology and motility. We find that TXNRD3 functions during epididymal sperm maturation in regulating chromatin organization and capacitation-associated mitochondrial activity. Biochemical analyses demonstrate that TXNRD3 protein levels diminish during sperm maturation, impairing redox homeostasis of head and flagellar proteins. Using ultrastructural analyses and live cell imaging, we reveal that mitochondria in *Txnrd3*^*−/−*^ sperm undergo structural defects and lose control of its activity especially during sperm capacitation. These findings provide insights into sperm redox regulation during epididymal maturation and its effect on capacitation-associated energy metabolism.

## Results

### *Txnrd3*^*−/−*^ sperm cells display defects in morphology and motility under capacitating conditions

**Figure 1:**
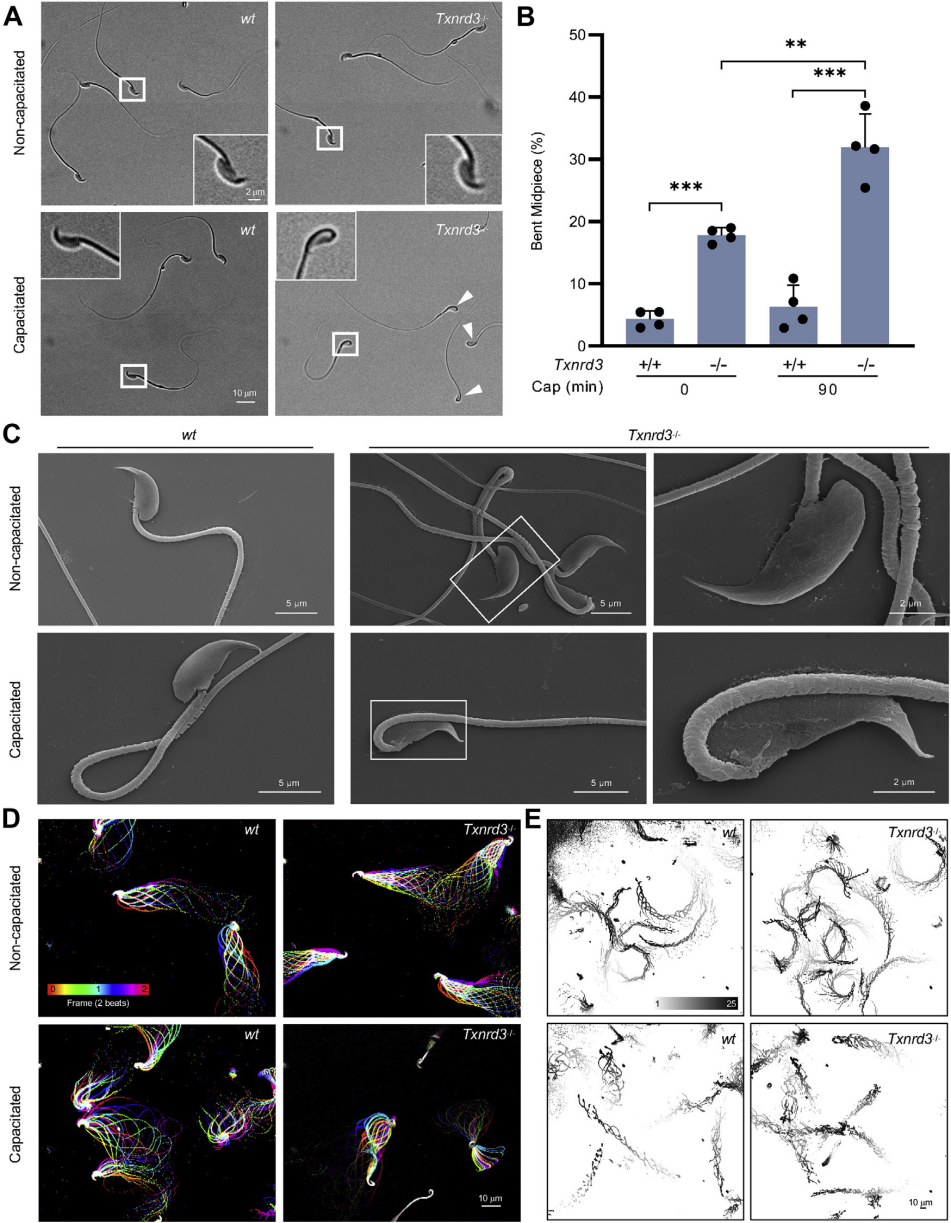
**TXNRD3-deficient sperm are morphologically abnormal with impaired hyperactivated motility.***A*, TXNRD3-deficient sperm exhibit abnormal bent midpiece following 90 min incubation under capacitation conditions. *B*, quantification of abnormal morphology from *A* (Mean ± SD, n = 4 each group. ∗∗*p* < 0.01, ∗∗∗*p* < 0.001). *C*, representative scanning electron microscopy (SEM) images of cauda epididymal sperm. *D*, aligned flagellar waveform traces. Movies were recorded at 200 fps: *wildtype* (*wt*) and *Txnrd3*^*−/−*^ sperm cells were attached on glass coverslips before (*top*) and after (*bottom*) 90 min capacitation. Overlays of flagellar traces from two beat cycles are generated by ImageJ. *E*, trajectory of free-swimming *wild type* and *Txnrd3*^*−/−*^ sperm in 0.3% methylcellulose at 0 min (*top*) and 90 min (*bottom*) after capacitation. Movies were taken at 50 fps for 2 s at 37 °C. Overlays of flagellar traces are generated by ImageJ. TXNRD3, thioredoxin-glutathione reductase.

In the accompanying study by Dou *et al.* (2022) (18), we showed that *Txnrd3*^*−/−*^ mice compromise fertility *in vivo* and sperm fertilizing ability *in vitro*. To understand why *Txnrd3*^*−/−*^ males exhibit sub-fertility *in vivo* and *in vitro* (18), we first examined sperm morphology and count from the cauda epididymis. The number of sperm produced by *Txnrd3*^*−/−*^ males was smaller than that from 2 to 3 months old heterozygous littermates (Fig. S1*A*). Among them, some *Txnrd3*^*−/−*^ sperm displayed bending within midpiece, specifically evident after 90 min incubation under capacitating conditions (Fig. 1, *A* and *B*), whereas sperm bent around annulus (*i.e.*, hairpin) were negligible in both *wildtype* and *Txnrd3*^*−/−*^ mice. Scanning electron microscopy analysis did not reveal any gross abnormalities in the sperm midpiece (Fig. 1*C*).

Prompted by the observation of capacitation-associated bending within midpiece of *Txnrd3*^*−/−*^ sperm, we next analyzed sperm motility. Flagellar waveform analysis revealed that *Txnrd3*^*−/−*^ sperm become stiff in the midpiece and beat abnormally after incubating under capacitating conditions (Figs. 1*D* and S1*B* and Video S1). Accordingly, CASA analysis found that overall motility of *Txnrd3*^*−/−*^ sperm was diminished (Fig. S1*C*). By contrast, hyperactivated motility was not significantly affected, likely because the *Txnrd3*^*−/−*^ sperm that are not bent still developed hyperactivated motility. To better understand the effect of TXNRD3 deficiency on free-swimming pattern, sperm cells were placed in 0.3% methylcellulose which mimics viscous environment in the female reproductive tract. Swimming trajectory traced by time-lapse video microscope demonstrated that *Txnrd3*^*−/−*^ sperm move a shorter distance at a given time after capacitation (Fig. 1*E* and Video S2), suggesting that the bending within midpiece prevents them from swimming as efficiently as *wildtype* sperm.

### Absence of TXNRD3 affects the redox state of both head and tail of sperm

**Figure 2:**
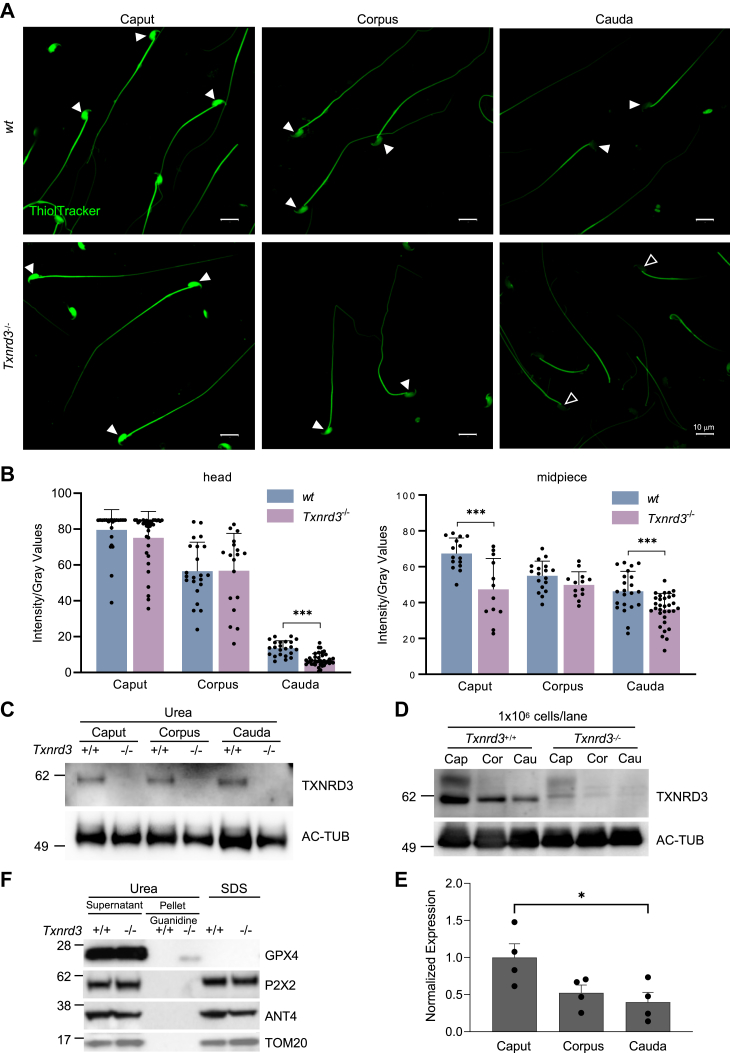
**Free thiol level and oxidative status are altered in *Txnrd3***^***−/−***^**spermatozoa.***A*, free thiol groups level in epididymal sperm from *wildtype* and *Txnrd3*^*−/−*^ mice. Spermatozoa were isolated from the caput, corpus and cauda epididymis, then stained with ThiolTracker. The intensity indicated the free thiol groups level in sperm. *Arrowheads* indicate the position of comparison, and *empty arrowheads* indicate absence/decrease of intensity. *B*, relative fluorescence intensity (*gray value*) of free thiol levels in sperm head and midpiece measured by ImageJ. *C*, immunoblotting analysis of TXNRD3 extracted from caput, corpus, and cauda sperm by urea. *D*, TXNRD3 is less soluble in SDS in concert with sperm maturation in the epididymis. Acetylated tubulin (AC-TUB) level was used for identical loading evaluation. *E*, quantification of TXNRD3 expression from *D*. *F*, immunoblotting analysis shows GPX4, a potential TXNRD3 substrate, exhibited stronger resistance to lysis buffer in the absence of TXNRD3. Other mitochondrial or midpiece localized proteins such as ANT4 (ADP/ATP translocase 4), P2X2 (P2X purinoceptor 2), and TOM20 (translocase of outer mitochondrial membrane 20) were well solubilized in all detergents tested. GPX4, glutathione peroxidase 4; TXNRD3, thioredoxin-glutathione reductase. Mean ± SD. ∗*p* < 0.05, ∗∗∗*p* < 0.001.

**Figure 3:**
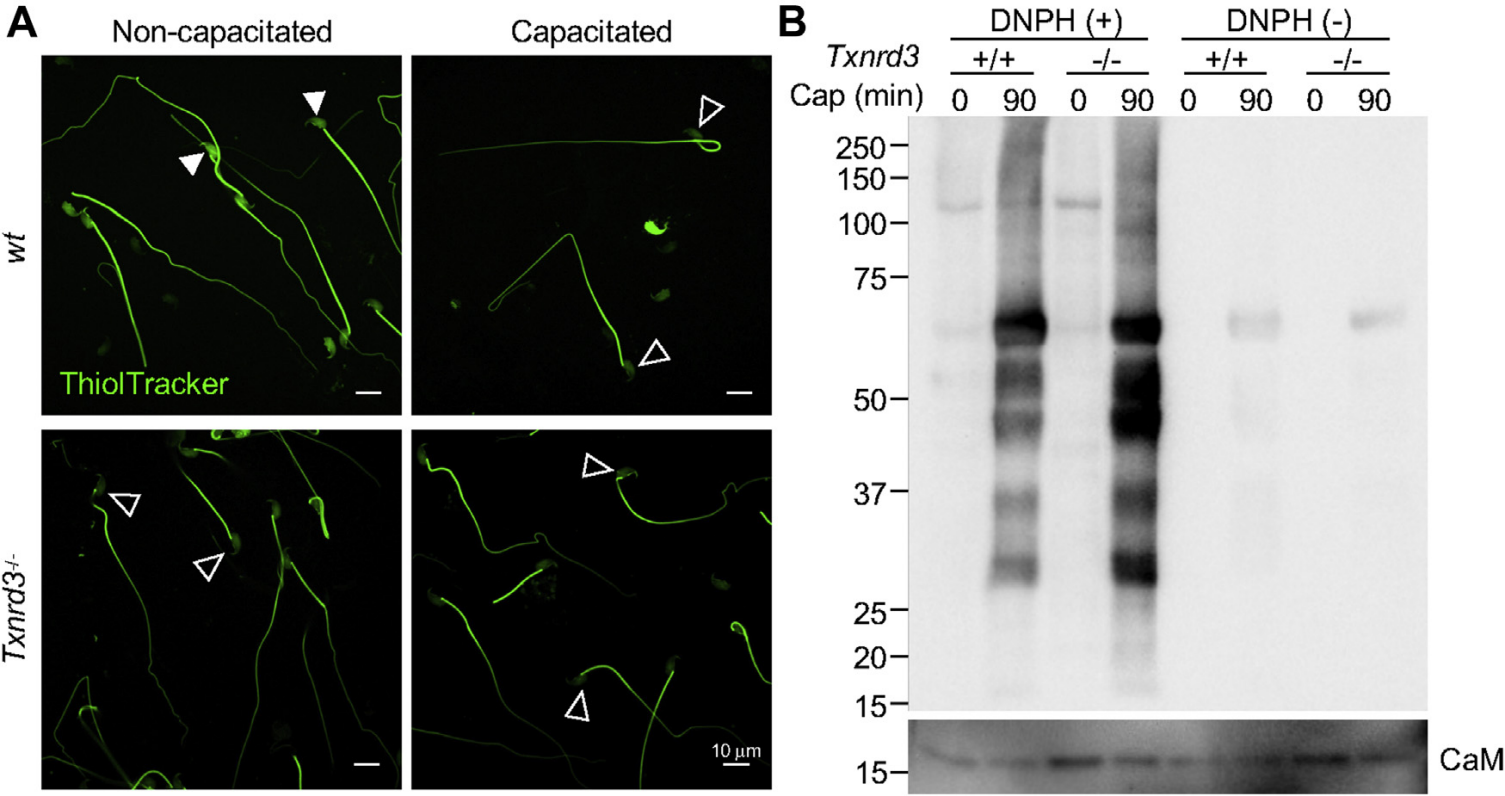
**TXNRD3-deficient sperm undergo more oxidation after capacitation.***A*, free thiol groups level revealed with ThiolTracker in cauda epididymal sperm from *wildtype* and *Txnrd3*^*−/−*^ mice before and after 90 min capacitation. *Arrowheads* indicate the position of comparison, and *empty arrowheads* indicate absence/decrease of intensity. *B*, protein carbonylation level in sperm before and after capacitation, indicates a higher oxidation in capacitated *Txnrd3*^*−/−*^ sperm. The carbonyl groups derivatized by DNPH (2,4-dinitrophenylhydrazine) were detected and quantified by western blotting using DNP antibody.

TXNRD3 is enriched in the testis but not readily detected in the epididymis (17). Our analysis of the publicly available scRNA-seq dataset of mouse testis (19) found that *Txrnd3* mRNA expresses gradually more from spermatogonia, spermatocytes, to early spermatids but the expression level is much reduced in late spermatids (Fig. S2). We hypothesized that diminishing TXNRD3 mRNA and protein levels during sperm development underlies the changes in the overall redox state of sperm proteins along the epididymal tract and subsequent capacitation of sperm. Thus, we examined the distribution and the extent of protein thiol modification in the absence of TXNRD3 by labeling epididymal sperm cells with ThiolTracker that reacts with reduced thiols (free “-SH”) irreversibly (Fig. 2, *A* and *B*). Consistent with the previously reported increasing disulfide bond formation in sperm during their transit along the male reproductive tract (20), *wildtype* sperm from corpus and cauda region displayed gradually diminishing fluorescence intensity, more noticeably in the head, whereas caput sperm exhibited strong fluorescence over the entire cell (Fig. 2, *A* and *B*). Using BODIPY-NEM, another thiol-reactive dye, we also found that, in the absence of TXNRD3, free thiol content is further decreased modestly, yet significantly, in cauda sperm (Fig. S3*A*). Loss of TXNRD3 did not affect the overall diminishing fluorescence patterns from caput to cauda. It is possible that other reductases function redundantly and compensate for the loss of TXNRD3. Indeed, both TXNRD1 and TXNRD2 proteins are equally detected in *wildtype* and *Txnrd3*^*−/−*^ sperm (Fig. S3*B*). In the absence of TXNRD3, however, free thiol content was significantly lower in the head of cauda sperm as well as in the midpiece of caput and cauda sperm than those of *wildtype* (Figs. 2, *A* and *B* and 3*A*, *arrowheads*).

We next examined whether changes in TXNRD3 protein levels resulted in more oxidized sperm proteins on thiols in cauda than caput. As the oxidized proteins in cauda sperm might be more resistant to be extracted in SDS lysis buffer likely due to more extensive cross-linking involving disulfide bond, we employed urea to solubilize sperm proteins. Under this condition, we found no significant difference in TXNRD3 protein levels from caput, corpus, and cauda sperm (Fig. 2*C*). Yet, TXNRD3 in sperm was indeed less soluble in SDS lysis buffer during their descent through epididymis (Fig. 2, *D* and *E*). This suggests that TXNRD3 itself is likely a substrate of thioredoxin system and is oxidized during epididymal maturation, affecting its enzymatic activity.

Sperm midpiece harbors compartmentalized mitochondria (21) (see also Fig. 1*C*). As *Txnrd3*^*−/−*^ sperm specifically exhibit the bending within the midpiece, next we tested whether the TXNRD3 loss-of-function affects the redox states of mitochondrial proteins more specifically. In the SDS extracted fraction, the protein level of glutathione peroxidase 4 (GPX4)—a mitochondrial structural protein and potential substrate of TXNRD3 (17, 22)—was much lower in the cauda sperm (Fig. S3, *C* and *D*) just as seen for TXNRD3 (Fig. 2, *D* and *E*). By contrast, urea readily extracted GPX4 even from cauda sperm (Fig. 2*F*, *supernatant*), indicating that GPX4 becomes gradually more oxidized during epididymal transit, therefore SDS-insoluble. Intriguingly, we observed a urea-insoluble GPX4 fraction in *Txnrd3*^*−/−*^ cauda sperm, but not in *wildtype*, when the pellet was further solubilized by guanidine following urea treatment (Fig. 2*E*, *pellet*). The altered thiol redox status of GPX4 might affect its function in reducing hydrogen peroxide and lipid peroxides against oxidative stress (23, 24). Other nonstructural but membrane proteins localized in sperm mitochondria or midpiece such as ANT4 (ADP/ATP translocase 4), P2X2 (P2X purinoceptor 2), TOM20 (translocase of outer mitochondrial membrane 20), and the other two thioredoxin reductases—TXNRD1 and TXNRD2—were well solubilized in all detergents tested (Figs. 2*F* and S3*B*). Taken together, our data clearly showed that TXNRD3 is involved in thiol redox regulation of sperm proteins during epididymal transition. In the accompanying manuscript (18), we provide further evidence that TXNRD3 reduces a broad range of substrates during epididymal sperm maturation.

### Deregulation of capacitation-associated oxidation in chromatin organization in *Txnrd3*^*−/−*^ sperm

It has been known that redox signaling is involved in sperm capacitation (5). We next examined how free thiol levels change during sperm capacitation. Under capacitating conditions, *wildtype* sperm isolated from cauda epididymis were less reactive with ThiolTracker, particularly in the head, whereas *Txnrd3*^*−/−*^ sperm was not reactive regardless (Fig. 3*A*, *arrowheads*). These results suggest that sperm proteins were more oxidized in the absence of TXNRD3 during sperm maturation, which remain relatively stable during capacitation. To test this idea, we examined protein oxidation levels by detecting carbonyl groups inserted into proteins. Incubating sperm under capacitating conditions dramatically increased sperm protein carbonylation in general, consistent with the idea of active oxidative reactions occur during capacitation. We observed that the protein oxidation level was further enhanced in *Txnrd3*^*−/−*^ sperm (Figs. 3*B* and S4*A*). The cellular oxidation during sperm capacitation might be associated with superoxide dismutase 1 decrease (Fig. S4*B*), consistent with the note that ROS level increases during sperm capacitation (25, 26). The identity of proteins subject to capacitation-associated oxidation remains to be determined.

**Figure 4:**
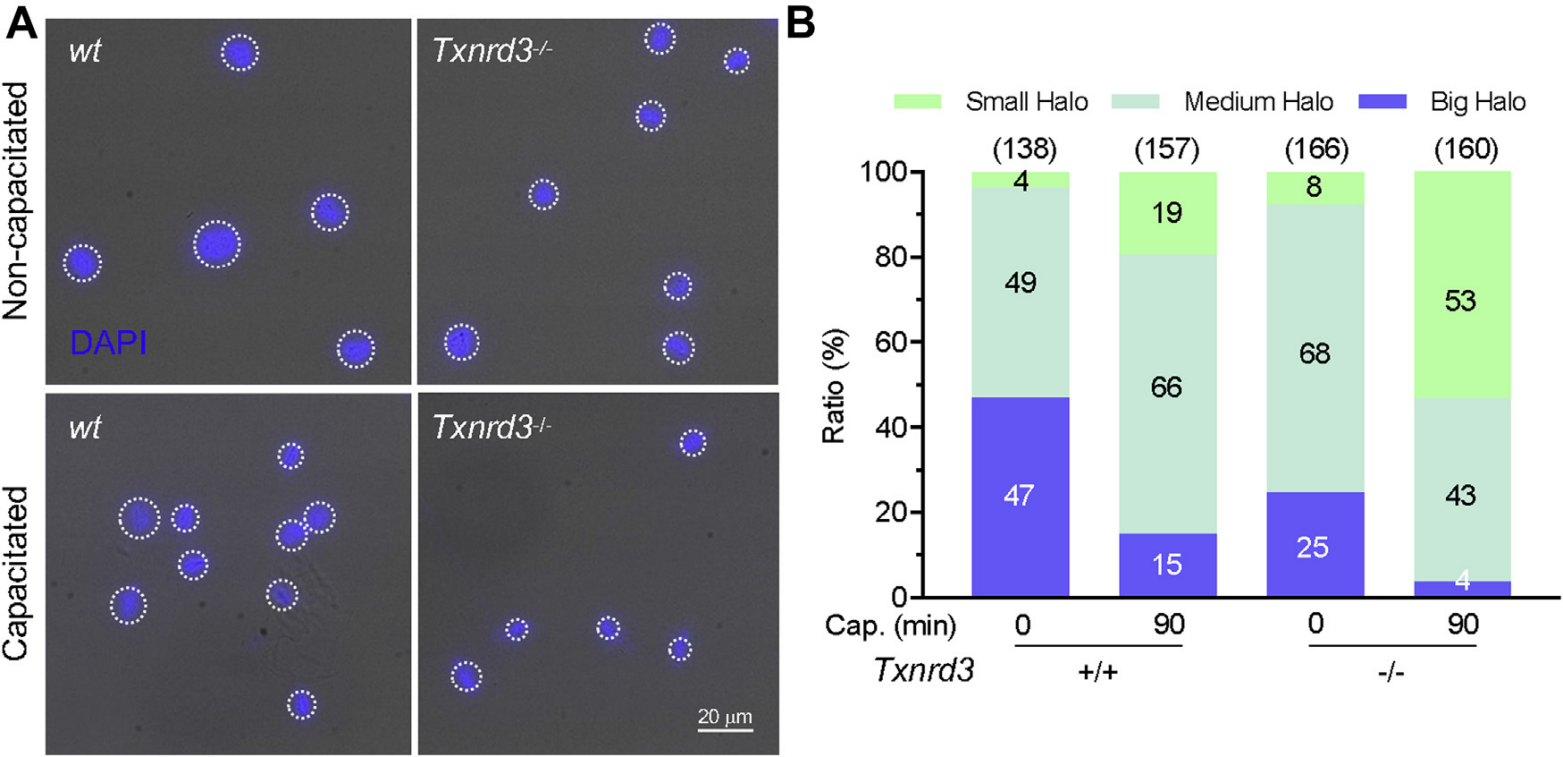
**TXNRD3-deficient sperm are more stable and resistant to acid denaturation after capacitation.***A*, sperm chromatin dispersion test evaluated smaller halo in *Txnrd3*^*−/−*^ sperm than that in *wildtype* sperm during capacitation, indicating extensive fragmented DNA after acid denaturation in *Txnrd3*^*−/−*^ sperm head. DNA was stained by DAPI (*blue*) after acid denaturation. *B*, quantification of halo sizes from *wild type* and *Txnrd3*^*−/−*^ sperm before and after capacitation. Based on sperm head size (∼8 μm), halo sizes are classified as small (<12 μm, ∼50% larger than head), medium (<17 μm, ∼100% larger), and big (>17 μm), respectively.

As the free thiol level changes were most dramatic in the head, we next investigated the loss of TXNRD3 function on sperm chromatin organization. We examined the extent of DNA integrity by halo assay where radial dispersion of the DNA fragments from the nucleus was measured upon artificial acid denaturation; the bigger the halo size is, the less damaged DNA fragment is present (27, 28, 29). Intriguingly, *Txnrd3*^*−/−*^ sperm resist the denaturation better, developing smaller halos than those of *wildtype* sperm (Fig. 4, *A* and *B*). This result suggests that more damaged, double-stranded DNA fragments are present in the nucleus in the absence of TXNRD3, likely due to more DNA fragments crosslinked to proteins. The nuclei of sperm incubated under capacitating conditions, developed smaller halos in both *wildtype* and *Txnrd3*^*−/−*^ sperm (Fig. 4, *A* and *B*). We further evaluated the altered resistance of DNA to acid denaturation by acridine orange (AO) assay; AO produces different emission when bound to single *versus* double stranded DNA (30, 31). We observed that double-stranded DNA was more stable and resistant to acid denaturation in both nucleus and mitochondria in capacitated *Txnrd3*^*−/−*^ sperm (Fig. S5). Taken together, the thiol oxidoreductase function is diminished in *Txnrd3*^*−/−*^ sperm compared to *wildtype* sperm. The data show that TXNRD3 supports redox regulation of epididymal sperm maturation, protecting mitochondrial structure and chromatin from oxidative damage during sperm capacitation.

### Lack of TXNRD3 dysregulates capacitation-induced remodeling of sperm mitochondrial ultrastructure

**Figure 5:**
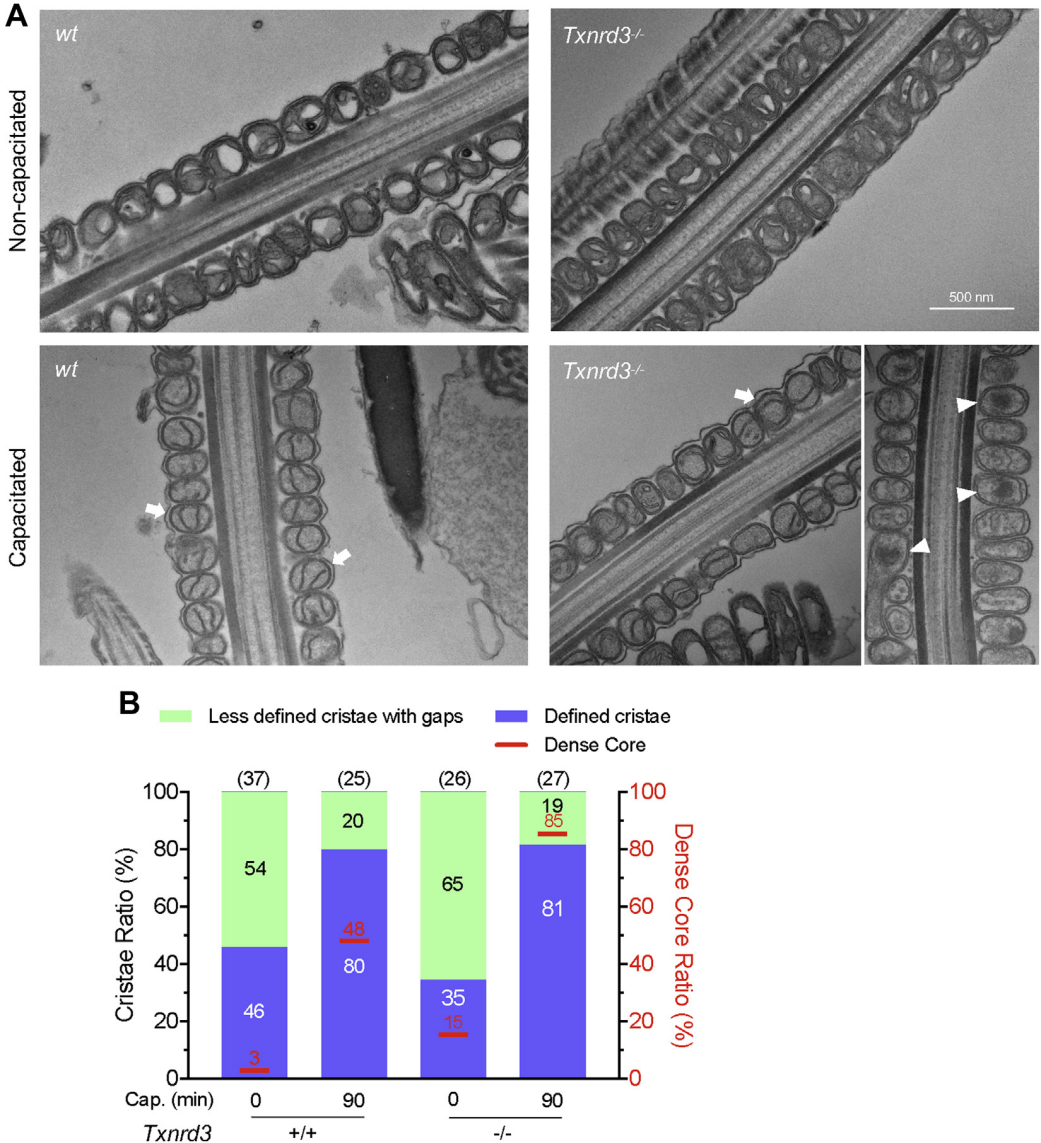
**Ultrastructural analysis of epididymal sperm.***A*, representative transmission electron microscopy (TEM) images of mitochondria from longitudinal sections of cauda epididymal sperm. *Arrows* indicate defined cristae found in mitochondria of capacitated sperm; *arrow heads* indicate dense cores found in mitochondria of *Txnrd3*^*−/−*^ sperm. *B*, classification of mitochondrial morphology in *wildtype* and *Txnrd3*^*−/−*^ sperm before and after capacitation. Total number of counted sperm are shown in *parentheses* above each bar. The *numbers* in the column indicate the proportion of each classification.

To maintain motility and support capacitation after ejaculation, mammalian sperm utilize nutrient molecules in the seminal plasma and in the female reproductive tract environment to generate energy (32). As terminally differentiated and highly polarized cells, sperm uniquely compartmentalize metabolic pathways in the flagella: the glycolytic enzymes are specifically localized in the principal piece, whereas mitochondrial oxidative phosphorylation (OXPHOS) occurs in the midpiece (21). A classic view on mouse sperm bioenergetics has been that glycolysis is the main metabolic pathway to support energy production for sperm motility although both glycolytic and OXPHOS are functional (33, 34, 35). Recent studies reported that oxygen consumption is accelerated in sperm incubated under capacitating conditions (36, 37), supporting increase of mitochondrial OXPHOS during capacitation. As mitochondria cristae organization—dimerization of the V-like shaped ATP synthase and its self-assembly into rows—determines respiratory efficiency (38, 39, 40, 41), we analyzed mitochondrial morphology by transmission electron microscopy. We found that mitochondria display more well-defined cristae after incubating under capacitating conditions in both *wildtype* and *Txnrd3*^*−/−*^ sperm (Fig. 5, *A* and *B*). In addition, we observed that capacitation induces electron-dense cores with few or no cristae in the mitochondrial matrix of capacitated sperm cells (Fig. 5, *A* and *B*). Notably, significantly a greater number of *Txnrd3*^*−/−*^ sperm displayed more mitochondrial condensation and collapsed cristae than *wildtype* sperm, which becomes more prominent in capacitated sperm. Thus, our results suggest that TXNRD3 deficiency compromises capacitation-associated mitochondrial dynamics and energetics, leading to energy imbalance, consistent with decreased sperm motility (Fig. S1).

### *Txnrd3*^*−/−*^ sperm lose control of mitochondrial membrane potential

**Figure 6:**
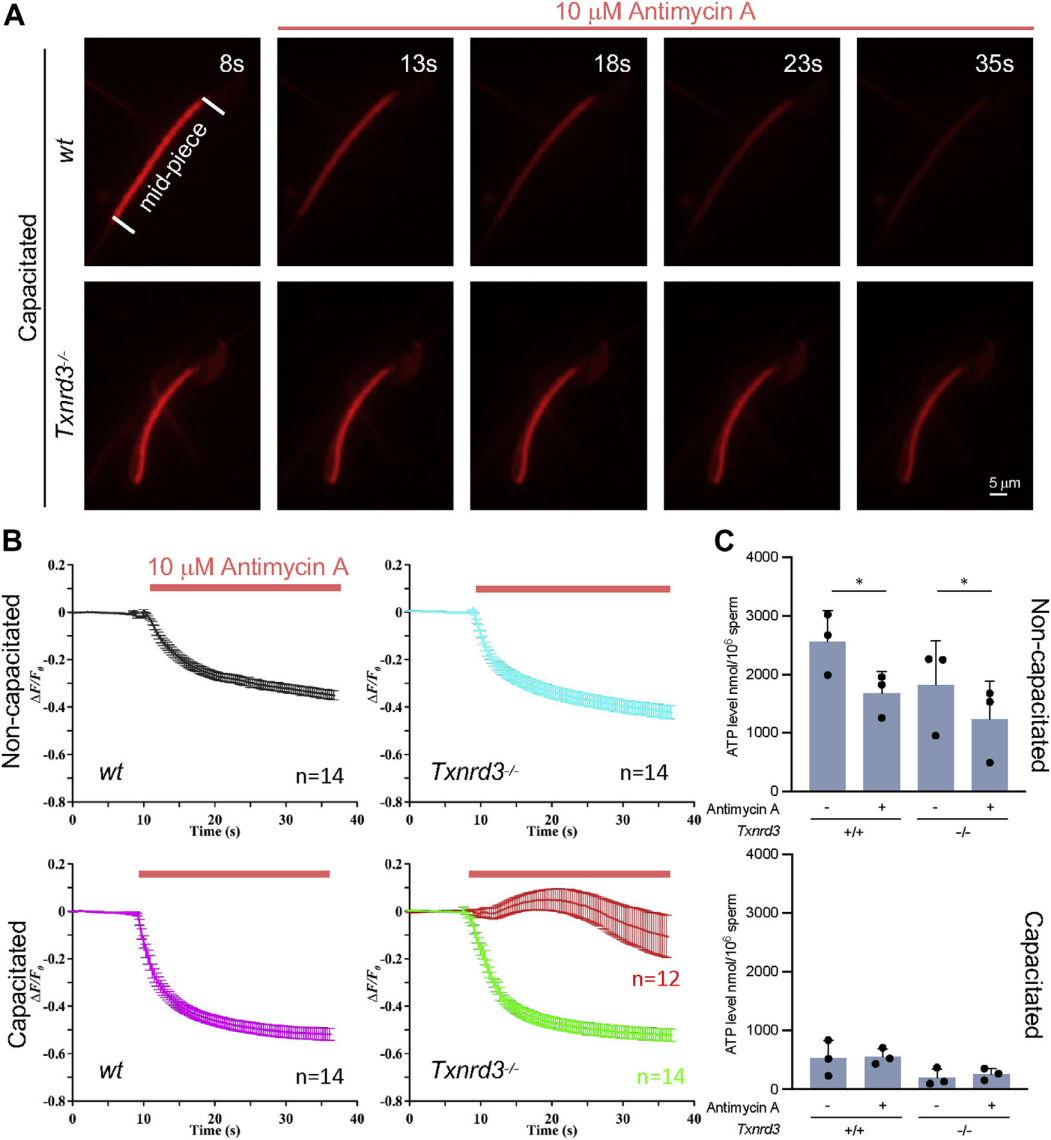
**Mitochondrial activity, indicated by membrane potential, is impaired in absence of TXNRD3.***A*, dynamic representative of mitochondrial membrane potential (ΔΨm) indicated by Mitotracker. Mouse sperm were capacitated then stained with 500 nM Mitotracker Deep Red which accumulates ΔΨm-dependently. ΔΨm was dissipated by antimycin A—an inhibitor of electron transport chain during oxidative phosphorylation. *B*, transition of the mitochondrial membrane potential. The changes of fluorescence intensity were calculated as ΔF/F_0_ (F_0_, the mean fluorescence intensity of the sperm midpiece before adding antimycin A (at 10 s); F, the fluorescence of the midpiece after adding antimycin A; ΔF = F − F_0_). Capacitated *Txnrd3*^*−/−*^ sperm show heterogeneous response to antimycin A, suggesting disrupted cellular respiration. *C*, ATP level from *wild type* and *Txnrd3*^*−/−*^ sperm before and after 90 min capacitation. 10 μM Antimycin A was added 30 min before sample collection. Statistical analyses between same genotype were performed using paired Student’s *t* test. Mean ± SD. ∗*p* < 0.05.

Mitochondrial membrane potential (ΔΨm) is an essential driving force for ATP synthesis and Ca^2+^ uptake (42). To better understand the loss-of-function effect of TXNRD3 on the capacitation-associated mitochondrial ultrastructure and sperm motility, we performed functional imaging to probe ΔΨm and measured ATP content. ΔΨm maintained by pumping hydrogen ions is coupled to the transfer of electrons through the electron transport chain (43). We observed that antimycin A, a potent electron transport chain inhibitor, dissipated ΔΨm probed by MitoTracker DeepRed (Fig. 6*A*, *wildtype*), a fluorescent dye which accumulates in mitochondria ΔΨm-dependently (44, 45, 46). Thus, the extent to which mitochondrial membrane is polarized can be compared with this functional yet indirect approach. Under noncapacitated conditions, ΔΨm is not significantly different between *wildtype* and *Txnrd3*^*−/−*^ sperm (Figs. 6*B* and S6). Under capacitating conditions, however, we found two distinct populations among TXNRD3-deficient sperm in terms of ΔΨm (Figs. 6 and S6); one group did not show any change in the fluorescent intensity of the dye, *i.e.*, an ΔΨm impervious to 10 μM antimycin A, suggesting an altered manner of regulating mitochondrial activity (*red trace*). By contrast, the other group of capacitated *Txnrd3*^*−/−*^ sperm exhibited ΔΨm at a similar level to that of capacitated *wildtype* sperm, which is more polarized than in noncapacitated cells. Intriguingly, ATP level is dramatically decreased in both *wildtype* and *Txnrd3*^*−/−*^ sperm after incubating under capacitating conditions (Fig. 6*C*, *compare noncapacitated versus capacitated*), which is in line with the direction of ΔΨm change during sperm capacitation. This suggests that ATP consumption is faster than synthesis to meet the energy demand for sperm capacitation (47) while both glycolysis and mitochondrial activity are accelerated (36, 37). We observed a trend toward the decrease of ATP level in *Txnrd3*^*−/−*^ sperm but found it not statistically significant (Fig. 6*C*), presumably due to a net result of two mixed sperm populations. Treatment of antimycin A reduced ATP level in noncapacitated sperm by 34% (*wildtype*) *versus* 35% (*Txnrd3*^*−/−*^) but had no effect on capacitated sperm (Fig. 6*C*), suggesting the potential adaptation in the contributions of mitochondria and glycolysis to ATP content during sperm capacitation. Taken together, these results suggest that the loss of TXNRD3 dysregulates the capacitation-associated mitochondrial activity in mouse sperm.

## Discussion

### Evidence that TXNRD3 supports sperm function and redox regulation in male fertility

The findings in this study demonstrate that the subfertility of *Txnrd3*^*−/−*^ sperm likely result from the combinatorial effects of defective chromatin organization and capacitation-induced tail bending within the midpiece. The high degree of sperm DNA fragmentation was reported to correlate with low fertilization rates, embryo quality, and pregnancy after IVF and intracytoplasmic sperm injection (28, 48). The bent tail would force the head backward, opposite to the swimming direction of the sperm. Additionally, TXNRD3 in sperm is gradually oxidized during epididymal transit, which is consistent with reduced free thiol group levels in cells along the epididymal tract. Therefore, the main timing of TXNRD3 function is during spermatogenesis in the testis and epididymal maturation rather than the subsequent fertilization process. Moderate changes in the free thiol levels and redox status of the *Txnrd3*^*−/−*^ sperm may be due to the increased level of thioredoxin 1 (18), which is a conserved thiol oxidoreductases and substrate of TXNRDs, and/or other thiol reductases such as TXNRD1 and TXNRD2 that could partly compensate for the loss of TXNRD3. Other thiol oxidoreductases specific to the testis and sperm such as thioredoxin domain–containing 2 (TXNDC2) (49, 50), thioredoxin domain–containing 3 (TXNDC3) (50, 51), and thioredoxin domain–containing 8 (TXNDC8) (52) remain to be examined. The data are also consistent with the mode wherein TXNRD3 supports accelerated redox remodeling during sperm maturation, which may also occur, albeit at a lower rate, in the absence of this enzyme. Consistent with the notion, the accompanying manuscript shows that overall, more oxidized proteins, based on their thiol status, were identified in the epididymal *Txnrd3*^*−/−*^ sperm including those of RNA binding-proteins and mitochondrial proteins involved in metabolism (18). Alternatively, the thiol redox status of certain proteins is marginally changed in the absence of TXNRD3, but the pattern of disulfide bond formation could be jumbled, sufficient to make *Txnrd3*^*−/−*^ sperm vulnerable to capacitation-associated oxidative damage.

### Mitochondrial function and control of metabolism during capacitation

Mitochondria is a key organelle still preserved in the fully differentiated spermatozoa unlike other cellular organelles. Previous knock-out studies of sperm-specific glyceraldehyde 3-phosphate dehydrogenase and phosphoglycerate kinase 2 have identified glycolysis as the main source of ATP to sustain the motility of mouse spermatozoa (53, 54). Because OXPHOS yields more ATP per mol of glucose than glycolysis, the discordant contribution of the two metabolic pathways to provide the energy for mouse sperm motility has been an unresolved puzzle for a long time. A comparative study revealed that sperm of closely related mouse species with higher oxygen consumption rate were able to produce higher amounts of ATP, achieving higher swimming velocities (55). Recent observations of capacitation-associated accelerated oxygen consumption in mouse sperm (36, 37) further support increased in mitochondrial activity for higher energy demand in sperm. Here we show, to our knowledge, that capacitation induces sperm mitochondria to form more defined cristae for the first time (Fig. 5). As ATPase dimerization and self-assembly drive cristae formation (38, 39, 40), this result further corroborates the idea that capacitation enhances OXPHOS pathway to supply additional ATP.

At the same time, we also made surprising but consistent observations with other groups: first, capacitating sperm cells display a more hyperpolarized ΔΨm than noncapacitated cells (Fig. 6*B*), which was also seen with a different dye (56). Second, capacitated sperm contains ATP to much less degree (Fig. 6*C*), suggesting that capacitation favors ATP consumption over synthesis (47). These results are seemingly incompatible with a decline of mitochondrial membrane potential coupled with ATP synthesis by F0-F1 ATP synthase (57) as well as capacitation-associated accelerated oxygen consumption (36, 37). It remains to be further investigated dynamics and relative partitioning of glycolysis *versus* mitochondrial contribution to sperm bioenergetics and the potential role of ΔΨm in regulating these processes.

Interestingly, we observed that a significant portion of TXNRD3-deficient sperm not only fail to control ΔΨm but also develop a more apparent electron-dense core in the mitochondrial matrix. As similar dense cores were previously observed in *Pmca4*^*−/−*^ sperm that might potentially experience Ca^2+^ overload (58), the TXNRD3-dependent redox signaling might impact on Ca^2+^ homeostasis during sperm capacitation. It requires further work to determine whether the TXNRD3-deficient sperm cells that lost control of ΔΨm are the same sperm populations with the electron-dense core and/or with deregulated Ca^2+^ homeostasis. Generation of a mouse model expressing a genetically encoded Ca^2+^ indicator specifically targeted to sperm mitochondria will help to directly address the functional relationship of redox regulation of mitochondria dynamics and Ca^2+^ homeostasis in sperm metabolism during capacitation.

In summary, our study investigated the TXNRD3 function and potential cellular mechanisms in sperm maturation and male fertility. The absence of TXNRD3 deregulates the redox control of sperm chromatin and mitochondrial structure proteins during capacitation, resulting in distinct bent midpiece and defective mitochondrial respiration; the loss-of-function prevents *Txnrd3*^*−/−*^ sperm from properly positioning to penetrate the egg and presumably also from efficiently producing ATP. *Txnrd3*^*−/−*^ mice are fertile in controlled laboratory environment without limitation of resource and male competition. Yet our findings from *in vitro* experiments show that TXNRD3 and the thioredoxin system intimately contribute to sperm function and male reproduction. This new knowledge could help to define conditions for assisted reproductive technology in clinic.

## Experimental procedures

### Mice

*Txnrd3*-null mice (18) are generated and maintained on a C57BL/6 background. Wildtype C57BL/6 mice were purchased from Charles River Laboratory. Mice were cared according to the guidelines approved by the Institutional Animal Care and Use Committee of Yale University (#20079).

### Mouse sperm preparation and *in vitro* capacitation

Epididymal spermatozoa from adult male mice were collected by swim-out from caudal epididymis in standard Hepes saline (HS) medium (in mM, 135 NaCl, 5 KCl, 1 MgSO_4_, 2 CaCl_2_, 20 Hepes, 5 glucose, 10 lactic acid, 1 Na pyruvate, pH 7.4 adjusted with NaOH, 320 mOsm/l) (59, 60). 2 × 10^6^ cells/ml sperm were incubated in human tubular fluid (HTF) medium (EMD Millipore) at 37 °C, 5% CO_2_ for 90 min to induce capacitation.

### Antibodies and reagents

Rabbit polyclonal antibodies specific to mouse TXNRD3 and GPX4 (17) were described previously. All the other antibodies used in this study are commercially available as follows: ANT4 (Abcam, ab136959), P2X2 (Santa Cruz, sc-12211), TOM20 (Santa Cruz, sc-11415), TXNRD1 (Bioss Antibodies, bs-8299R), TXNRD2 (Proteintech, 16360-1-AP), and acetylated tubulin (Sigma, T7451). All chemicals were from Sigma Aldrich unless otherwise indicated.

### Single-cell RNA-seq analysis

The raw count matrice mouse (GSE109033) testis single cell RNA (scRNA)-seq datasets (61) was downloaded from Gene Expression Omnibus (GEO) database (https://www.ncbi.nlm.nih.gov/geo/). The downloaded raw count matrices were processed for quality control using the Seurat package (ver.3.2.3) as previously described (59, 62). Briefly, cells with less than 200 expressed features, higher than 9000 (GSE109033) or 10,000 (GSE109037) expressed features and higher than 20% (GSE109037) or 25% (GSE109033) mitochondrial transcript fraction were excluded to select single cells with high quality mRNA profiles. The data were normalized by the total expression, scaled, and log transformed. Identification of 2000 highly variable features was followed by PCA to reduce the number of dimensions representing each cell. Statistically significant 15 PCs were selected based on the JackStraw and Elbow plots and provided as input for constructing a K-nearest-neighbors graph based on the Euclidean distance in PCA space. Cells were clustered by the Louvain algorithm with a resolution parameter 0.1. Uniform Manifold Approximation and Projection was used to visualize and explore cluster data. Marker genes that define each cluster were identified by comparing each cluster to all other clusters using the MAST (63) provided in Seurat package. In order to correct batch effects among samples and experiments, we applied the Harmony package (ver.1.0) (64) to the datasets. The Markov Affinity-based Graph Imputation of Cells algorithm (ver.2.0.3) (65) was used to denoise and the count matrix and impute the missing data. In these testis datasets from adult human, we identified 23,896 high quality single cells, that were clustered into seven major cell types, including spermatogonia, spermatocytes, early spermatids, late spermatids, peritubular myoid cell, endothelial cell, and macrophage. Similarly, we exploited 30,268 high quality single cells from eight adult and three 6-days postpartum mouse testis tissue samples and subsequently defined 11 major cell populations.

### Sperm immunocytochemistry and free thiol labeling assay

As described previously (59, 66), mouse sperm were washed in PBS twice, attached on the glass coverslips, and fixed with 4% paraformaldehyde (PFA) in PBS at room temperature (RT) for 10 min. Fixed samples were permeabilized using 0.1% Triton X-100 in PBS at RT for 10 min, washed in PBS, and blocked with 10% goat serum in PBS at RT for 1 h. Cells were stained with antibody in PBS supplemented with 10% goat serum at 4 °C overnight. After washing in PBS, the samples were incubated with goat anti-rabbit Alexa 647 or Alexa 555-plus (Invitrogen, 1:1000) in 10% goat serum in PBS at RT for 1 h. Hoechst dye was used to stain nucleus to indicate sperm head. For BODIPY-N-ethylmaleimide labeling, reduced thiols within proteins were alkylated with BODIPY FL maleimide (final concentration of 10 nM, ThermoFisher) for 30 min in the dark after permeabilization. The sample was then quenched by the addition of 500 mM 2-mercaptoethanol for 30 min in the dark, followed by three times washing in PBS. For ThiolTracker (ThermoFisher) labeling, fixed sperm were stained with 20 μM dye, followed by three times washing in PBS. Stained sperm samples were mounted with Prolong gold (Invitrogen) and cured for 24 h, followed by imaging with Zeiss LSM710 Elyra P1 using Plan-Apochrombat 63×/1.40 or alpha Plan-APO 100×/1.46 oil objective lens (Carl Zeiss).

### Protein extraction and Western blotting

Whole sperm protein was extracted as previously described (66, 67, 68). In short, mouse epididymal spermatozoa (1 × 10^6^ cells) washed in PBS were directly lysed in 20 μl 2× SDS sample buffer or 8 M urea. The whole sperm lysate was centrifuged at 15,000*g*, 4 °C for 10 min. Supernatant were adjusted to 50 mM DTT and denatured at 95 °C for 10 min before loading to a SDS-PAGE gel. The proteins were transferred on a nitrocellulose membrane. The membrane was incubated overnight at 4 °C with primary antibody, followed by washing with PBST and incubation with appropriate secondary antibody for 1 h. Bands were visualized using chemiluminescence and imaged by ChemiDoc (Bio-Rad). Primary antibodies used for Western blotting were rabbit anti-mouse TXNRD3 (1:2000), GPX4 (1:2000), ANT4 (1 μg/ml), P2X2 (1 μg/ml), TOM20 (0.2 μg/ml), TXNRD1 (1 μg/ml), TXNRD2 (1 μg/ml), and mouse anti-acetylated tubulin (1: 20,000). Secondary antibodies were anti-rabbit IgG-HRP (1:10,000) and anti-mouse IgG-HRP (1:10,000) from Jackson ImmunoResearch. Normalized expression was measured by ImageJ software (v1.53).

### Protein oxidation

Carbonyl groups inserted into proteins by oxidative reactions were evaluated with protein carbonyl assay kit (Abcam, ab178020) (56, 69). Briefly, samples were homogenized and processed according to the manufacturer’s protocol. The carbonyl groups in the solubilized protein samples were derivatized using DNPH (2,4 dinitrophenyl hydrazine) or control solution for 15 min and then neutralized. The samples were then loaded onto SDS-PAGE gels, and DNP-conjugated proteins were detected by western blotting using primary DNP antibody and HRP-conjugated secondary antibody.

### Flagella waveform analysis

To tether sperm head for planar beating, noncapacitated or capacitated spermatozoa (2–3 × 10^5^ cells) from adult male mice were transferred to the fibronectin-coated 37 °C chamber for Delta T culture dish controller (Bioptechs) filled with HS and Hepes-buffered HTF medium (H-HTF) (68), respectively. Flagellar movements of the tethered sperm were recorded for 2 s with 200 fps using pco.edge sCMOS camera equipped in Axio observer Z1 microscope (Carl Zeiss). All movies were taken at 37 °C within 10 min after transferring sperm to the imaging dish. ImageJ software (v1.53) (70) was used to measure beating frequency of sperm tail and to generate overlaid images to trace waveform of sperm flagella as previously described (68).

### Sperm motility analysis

Aliquots of sperm were placed in slide chamber (CellVision, 20 mm depth) and motility was examined on a 37 °C stage of a Nikon E200 microscope under 10× phase contrast objective (CFI Plan Achro 10×/0.25 Ph1 BM, Nikon). Images were recorded (40 frames at 50 fps) using CMOS video camera (Basler acA1300-200 μm, Basler AG) and analyzed by computer-assisted sperm analysis (CASA, Sperm Class Analyzer version 6.3, Microptic). Sperm total motility and hyperactivated motility was quantified simultaneously. Over 200 motile sperm were analyzed for each trial, at least three biological replicates were performed for each genotype. To track swimming trajectory, the sperm motility was videotaped at 50 fps. The images were analyzed using ImageJ software (v1.53) (70) by assembling overlays of the flagellar traces generated by hyperstacking binary images of 20 frames of 2 s movies coded in a gray intensity scale.

### Scanning electron microscopy

As previously described (59), sperm cells were attached on the glass coverslips and fixed with 2.5% glutaraldehyde in 0.1 M sodium cacodylate buffer (pH 7.4) for 1 h at 4 °C and post fixed in 2% osmium tetroxide in 0.1 M cacodylate buffer (pH 7.4). The fixed samples were washed with 0.1 M cacodylate buffer for three times and dehydrated through a series of ethanol to 100%. The samples were dried using a 300 critical point dryer with liquid carbon dioxide as transitional fluid. The coverslips with dried samples were glued to aluminum stubs and sputter coated with 5 nm platinum using a Cressington 208HR (Ted Pella) rotary sputter coater. Prepared samples were imaged with Hitachi SU-70 scanning electron microscope (Hitachi High-Technologies).

### Transmission electron microscopy

Collected epididymal sperm cells were washed and pelleted by centrifugation and fixed in 2.5% glutaraldehyde and 2% PFA in 0.1 M cacodylate buffer pH 7.4 for 1 h at RT. Fixed sperm pellets were rinsed with 0.1 M cacodylate buffer and spud down in 2% agar. The chilled blocks were trimmed, rinsed in the 0.1 M cacodylate buffer, and replaced with 0.1% tannic acid in the buffer for 1 h. After rinsing in the buffer, the samples were postfixed in 1% osmium tetroxide and 1.5% potassium ferrocyanide in 0.1 M cacodylate buffer for 1 h. The postfixed samples were rinsed in the cacodylate buffer and distilled water, followed by *en bloc* staining in 2% aqueous uranyl acetate for 1 h. Prepared samples were rinsed and dehydrated in an ethanol series to 100%. Dehydrated samples were infiltrated with epoxy resin Embed 812 (Electron Microscopy Sciences), placed in silicone molds, and baked for 24 h at 60 °C. The hardened blocks were sectioned in 60-nm thickness using Leica UltraCut UC7. The sections were collected on grids coated with formvar/carbon and contrast stained using 2% uranyl acetate and lead citrate. The grids were imaged using FEI TecnaiBiotwin Transmission Electron Microscope (FEI) at 80 kV. Images were taken using MORADA CCD camera and iTEM (Olympus) software. The sperm are classified based on the leading proportion of defined cristae (defined cristae, >50%; less defined cristae with gaps, <50%). Sperm are classified as dense cored sperm when more than one mitochondrion with dense core is observed per sperm.

### Sperm chromatin dispersion test (halo assay)

Halo assay was modified according to a previous report (29). In brief, samples were diluted to the concentration of 0.5 to 1 × 10^7^ cells/ml, then mixed with low-melting-point aqueous agarose to obtain a final concentration of 0.7% agarose. 50 μl mixture was dispensed onto a glass slide precoated with 0.65% standard agarose, then covered by a coverslip for solidification at 4 °C for 4 min. The coverslip was then removed carefully before the sample was immersed into freshly prepared acid denaturation solution (0.08 N HCl) for 7 min, neutralizing and lysing solution 1 (0.4 M Tris-HCl, 0.8 M DTT, 1% SDS, 50 mM EDTA, pH 7.5) for 10 min, and neutralizing and lysing solution 2 (0.4 M Tris-HCl, 2 M NaCl, 1% SDS, pH 7.5) for 5 min at RT in sequence. The slide was then transferred to Tris-borate EDTA solution (90 mM Tris-borate, 2 mM EDTA, pH 7.5) for 2 min, then dehydrated in sequential 70%, 90%, and 100% EtOH (2 min each). After drying, the slide was stained with DAPI (2 μg/ml), then examined under microscope as described above. The Halo size (diameter) was defined and classified into big (>17 μm), medium (between 12 and 17 μm) and small size (<12 μm).

### Acridine orange assay

Staining was performed, as described previously (30, 31) with minor modification. Briefly, sperm cells were smeared on glass slide until dry, followed by washing in PBS pH 7.2. 4% PFA was used to fix cells for 15 min at RT, then covered by methanol for 5 min washed with PBS for 5 min. The sample was incubated in RNAase solution at 37 °C for 30 min. Washing with PBS of pH 7.2 for 5 min was performed between each step. 0.1 M HCl was used for DNA denaturation for 30 to 45 s, followed by AO staining working solution for 2 min. The slide was ready for examination under fluorescence microscopy after washing gently and drying.

### Mitochondrial membrane potential (ΔΨm) measurement

Epididymal sperm were attached on Delta T culture chamber coated with poly-D-Lysine (2 mg/ml) or together with Cell-Tak for 30 min (71). Unattached sperm were removed by the gentle pipette wash. Mitotracker deep red (working concentration 500 nM, ThermoFisher) was loaded in sperm for 30 min, followed by gentle rinse with HS or Hepes-buffered HTF medium H-HTF. All movies were taken at 37 °C by the pco.edge sCMOS camera equipped in Axio observer Z1 microscope (Carl Zeiss). The data were analyzed by Zen (Blue).

### ATP measurement

1 to 1.5 × 10^6^ sperm were left in HS as noncapacitated or incubated in HTF under capacitating conditions for 1 h, followed by 10 μM antimycin A or vehicle treatment for 30 min. Sperm suspensions were shock frozen in liquid nitrogen, ATP was then extracted by boiling for 10 min (53, 72). After cooling down on ice, the suspensions were centrifuged for 5 min at 20,000*g*, and the supernatants were collected. ATP levels were measured in triplicate samples on 96-well plates with the luciferase-based ATP bioluminescent assay kit (ThermoFisher, A22066) according to the manufacturer’s protocol by a luminometer (Tecan Infinite M1000).

### Quantification and statistical analysis

Statistical analyses were performed using unpaired Student’s *t* test by GraphPad Prism (v9.2) unless otherwise indicated. Differences were considered significant at ∗*p* < 0.05, ∗∗*p* < 0.01, and ∗∗∗*p* < 0.001.

## Data availability

All data are contained within the article and supporting information.

## Supporting information

This article contains supporting information.

## Conflict of interest

The authors declare that they have no conflicts of interest with the contents of this article.
