# Supplementary material for: Redox regulation by TXNRD3 during epididymal maturation underlies capacitation-associated mitochondrial activity and sperm motility in mice
Source: J Biol Chem. 2022 May 25;298(7):102077. doi: 10.1016/j.jbc.2022.102077 (PMC9218152; doi:10.1016/j.jbc.2022.102077)
Supplement: Supplemental Figures S1–S6 [file mmc3.docx]

**Redox regulation by TXNRD3 during epididymal maturation underlies capacitation-associated mitochondrial activation and sperm motility in mice**

**Huafeng Wang^1^, Qianhui Dou^2^, Kyung Jo Jeong^3^, Jungmin Choi^3,4^, Vadim N. Gladyshev^2^, Jean-Ju Chung^1,5*^**

^1^Departmet of Cellular and Molecular Physiology, Yale School of Medicine, New Haven, CT, United States

^2^Division of Genetics, Department of Medicine, Brigham and Women’s Hospital, Harvard Medical School, Boston, MA, United States

^3^Department of Biomedical Sciences, Korea University College of Medicine, Seoul, South Korea

^4^Department of Genetics, Yale School of Medicine, Yale University, New Haven, CT, United States

^5^Department of Obstetrics, Gynecology, and Reproductive Sciences, Yale School of Medicine, New Haven, CT, United States

*** Correspondence:** Jean-Ju Chung ([jean-ju.chung@yale.edu](mailto:jean-ju.chung@yale.edu))

Materials included:

Supplemental Figure 1

Supplemental Figure 2

Supplemental Figure 3

Supplemental Figure 4

Supplemental Figure 5

Supplemental Figure 6

Supplemental Video 1

Supplemental Video 2


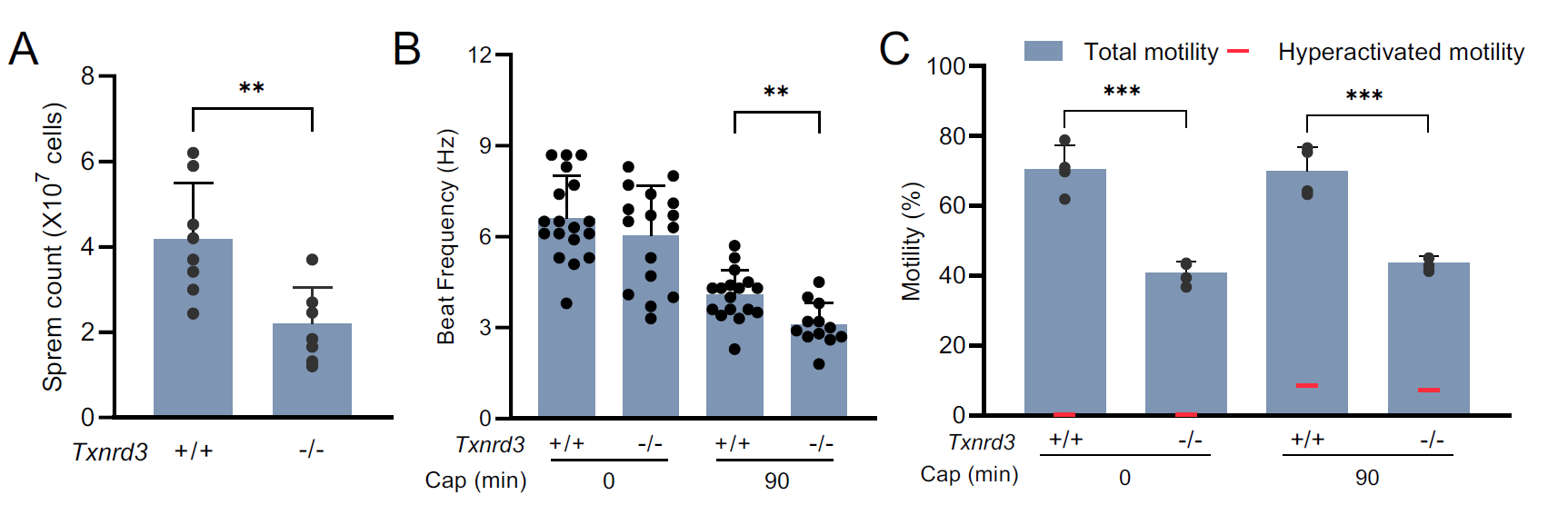


**Supplemental Figure 1.** **TXNRD3-deficient mice exhibit reduced sperm count and motility.** *A*, epididymal sperm count from littermates at ages of 3-4 months. *B*, flagellar beat frequency of *wild type* and *Txnrd3*^-/-^ sperm before (0 min, *wild type*, 6.6 ± 1.3 Hz; *Txnrd3*^-/-^, 6.0 ± 1.6 Hz) and after (90 min, *wild type*, 4.1 ± 0.8 Hz; *Txnrd3*^-/-^, 3.1 ± 0.7 Hz) capacitation. *C*, sperm total motility and hyperactivated motility before and after 90 min incubation under capacitation conditions. Mean ± SD. ***p* < 0.01, ****p* < 0.001.

**
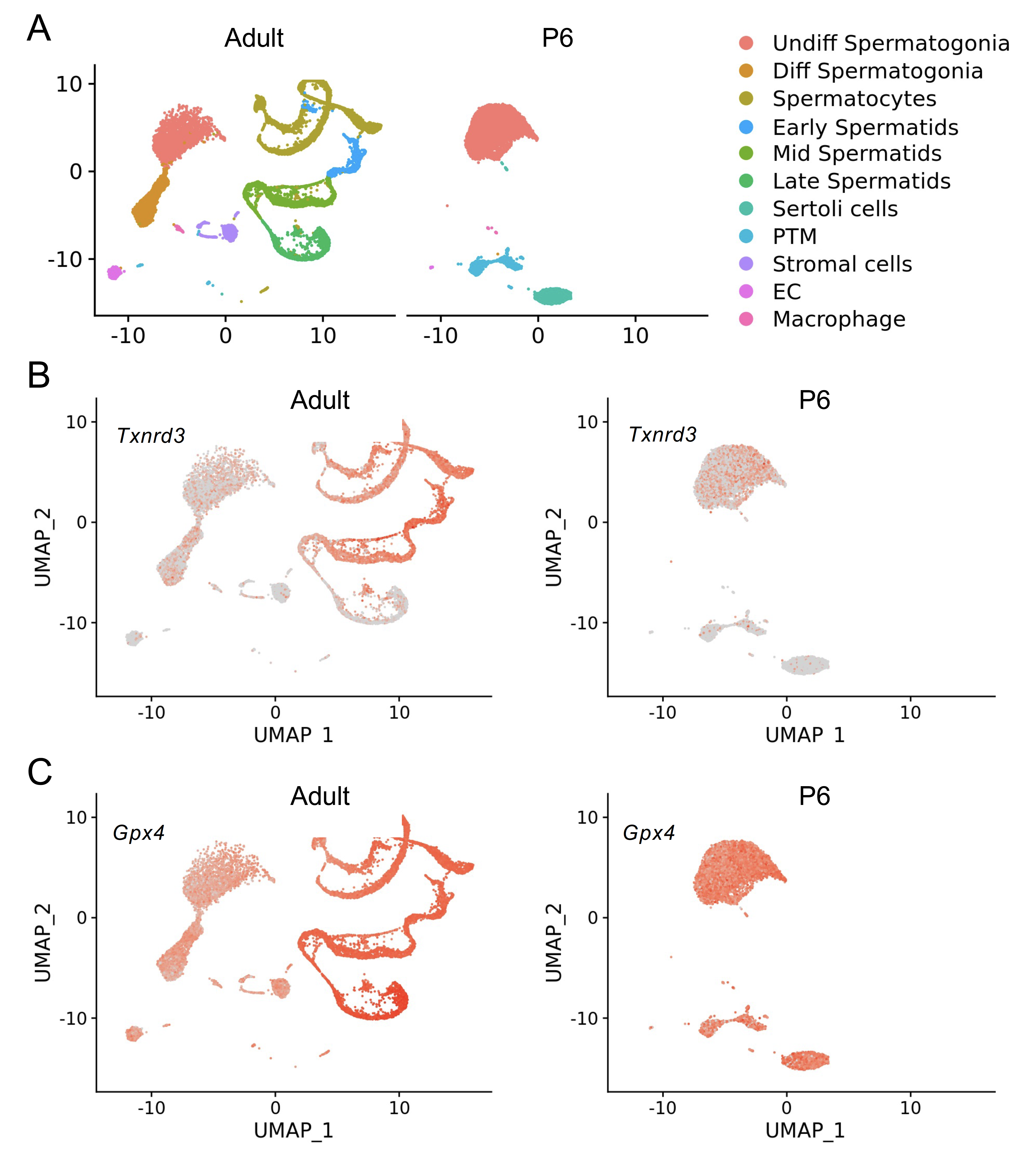
**

**Supplemental Figure 2. Single testicular cell transcriptome profiling from adult and postnatal day 6 (P6) mice.** *A*, clustering analysis of combined single-cell transcriptome data from testes. Each dot represents a single cell and is colored according to its cluster identity as indicated on the figure key. 11 cluster identities were assigned based on marker gene expression. PTM, Peritubular myoid; EC, Testicular endothelial cells. *B*, expression patterns of *Txnrd3* which is highly expressed in spermatogonia and spermatocytes but much less in spermatids. Red indicates high expression and gray indicates low or no expression. *C*, expression patterns of *Gpx4,* which is abundant through spermatogenesis.



**Supplemental Figure 3. Free thiol level and level of GPX4 from caput to cauda sperm.** *A*, spermatozoa isolated from the caput, corpus and cauda epididymis were stained with Bodipy-labeled NEM, followed by quenching with β-mercaptoethanol. Free thiol groups levels in the cells were visualized and compared each other under confocal microscope. Arrowheads indicate the position of comparison, and empty arrowheads indicate absence/decrease of intensity. *B*, TXNRD1 and TXNRD2 show same level in both *wild type* and *Txnrd3*^-/-^ sperm. *C*, GPX4 shows descending level in sperm during epididymal transit. *D*, localization of GPX4 in *wild type* and *Txnrd3*^-/-^ sperm.


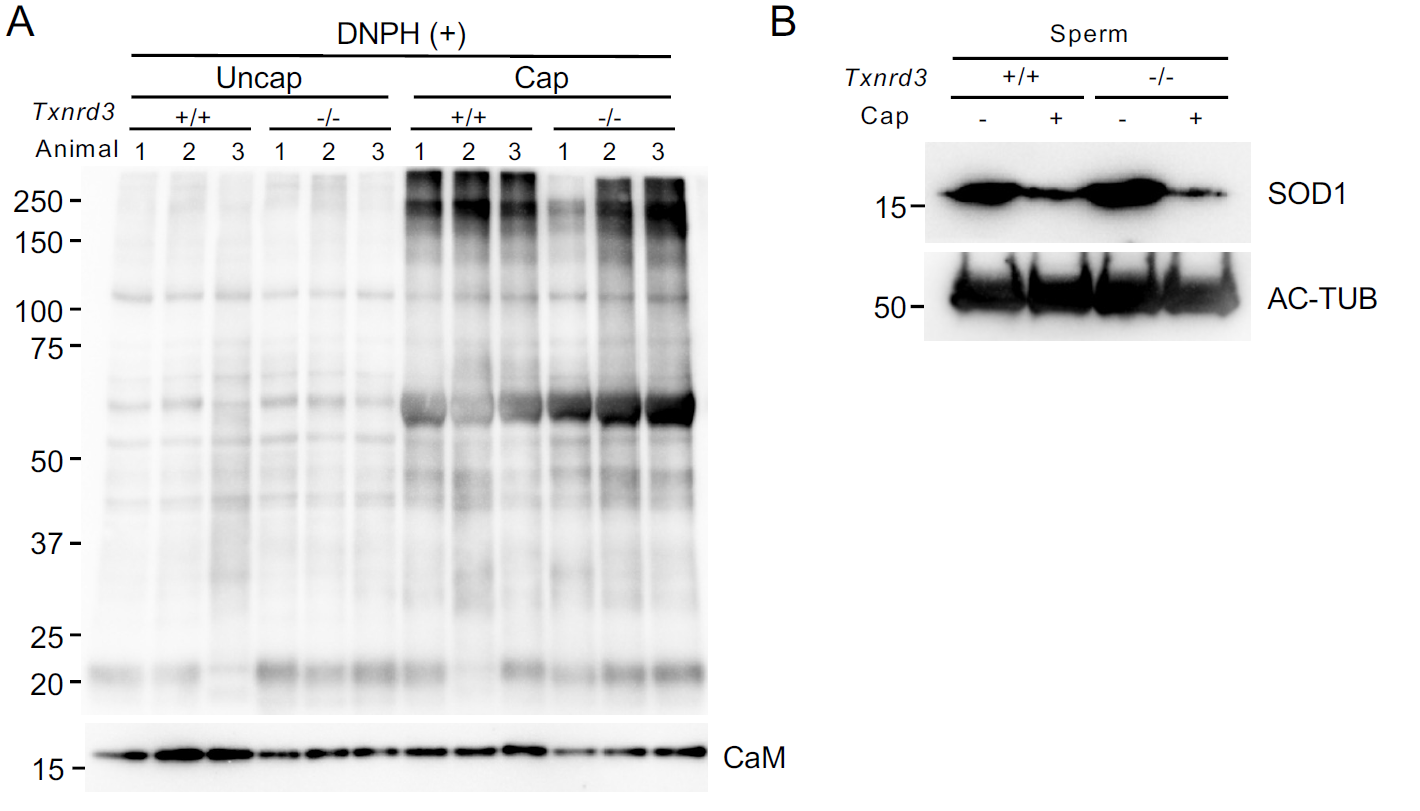


**Supplemental Figure 4. Protein oxidation status probed by protein carbonylation level is changed during sperm capacitation.** *A*, Protein carbonylation level was detected in *wild type* and *Txnrd3^-/-^* sperm before and after capacitation from multiple mice. The carbonyl groups derivatized by DNPH (2,4-dinitrophenylhydrazine) were detected and quantified by western blotting using DNP antibody. *B*, SOD1 shows decreased level during sperm capacitation but no obvious difference between wild type and *Txnrd3^-/-^* sperm before and after capacitation, respectively.


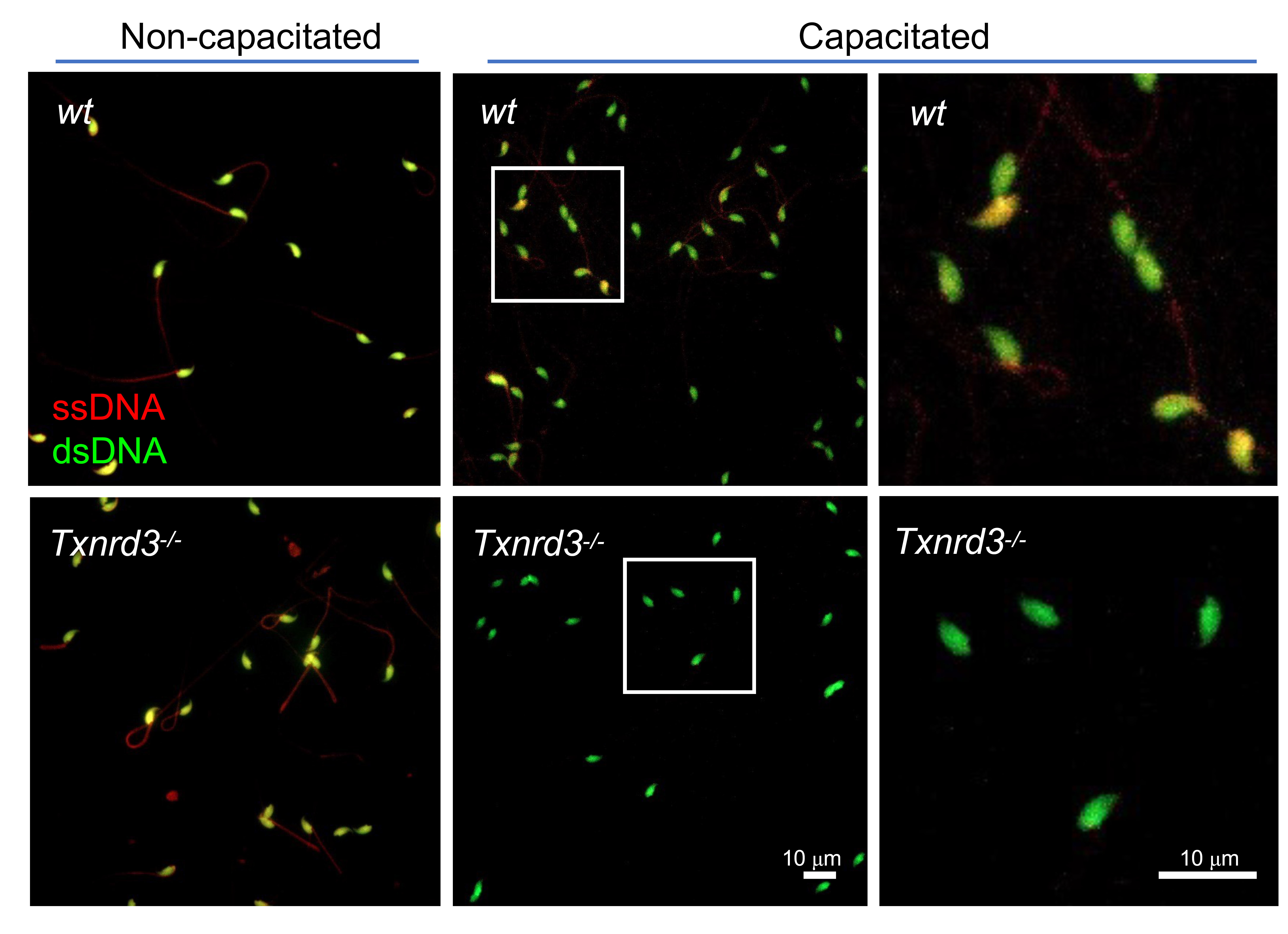


**Supplemental Figure 5.** **Evaluation of DNA state by Acridine Orange assay.** Sperm were stained with acridine orange staining working solution after HCl denaturation. Single-stranded DNA is visualized in Red, double-stranded DNA is in Green. *Txnrd3*^-/-^ sperm generated less single-stranded DNA after capacitation, indicating more resistance to acid denaturation.



**Supplemental Figure 6.** **Individual traces of mitochondrial membrane potential transition from *wild type* and *Txnrd3*^-/-^ sperm.** The traces of individual sperm cells are presented. The mitochondrial membrane potential was indicated by Mitotracker Deep Red, then dissipated by antimycin A - an inhibitor of electron transport chain. The dissipation amplitude suggested the mitochondrial function capacity. The changes of fluorescence intensity were calculated as ΔF/F_0_ (F_0_, the mean fluorescence intensity of the sperm midpiece before adding Antimycin A (at 10 s); F, the fluorescence of the midpiece after adding antimycin A; ΔF=F-F_0_). Capacitated *Txnrd3*^-/-^ sperm show heterogeneous response to antimycin A, suggesting disrupted cellular respiration.

**Supplemental Video 1. Head-tethered sperm from *wild type* and *Txnrd3*^-/-^ mice before and after incubating under capacitation conditions.**

**Supplemental Video 2. Free-swimming sperm from *wild type* and *Txnrd3*^-/-^ mice before and after incubating under capacitation conditions.**
